# Efficacy of *Lactobacillus* Administration in School-Age Children with Asthma: A Randomized, Placebo-Controlled Trial

**DOI:** 10.3390/nu10111678

**Published:** 2018-11-05

**Authors:** Chian-Feng Huang, Wei-Chu Chie, I-Jen Wang

**Affiliations:** 1 Institute of Epidemiology and Preventive Medicine, College of Public Health, National Taiwan University, Taipei 10055, Taiwan; davidbee416@gmail.com (C.-F.H.); weichu@ntu.edu.tw (W.-C.C.); 2 Taoyuan Psychiatric Center, Ministry of Health and Welfare, Taoyuan 33058, Taiwan; 3 Department of Pediatrics, Taipei Hospital, Ministry of Health and Welfare, No. 127, Su-Yuan Road, Hsin-Chuang Dist., Taipei 242, Taiwan; 4 College of Public Health, China Medical University, Taichung 40402, Taiwan; 5 School of Medicine, National Yang-Ming University, Taipei 112, Taiwan

**Keywords:** *Lactobacillus*, probiotics, asthma, Childhood Asthma Control Test, peak expiratory flow rate, immunoglobulin E

## Abstract

Probiotics may have immunomodulatory effects. However, these effects in asthma remain unclear and warrant clinical trials. Here, we evaluated the effects of *Lactobacillus paracasei* (LP), *Lactobacillus fermentum* (LF), and their combination (LP + LF) on the clinical severity, immune biomarkers, and quality of life in children with asthma. This double-blind, prospective, randomized, placebo-controlled trial included 160 children with asthma aged 6–18 years (trial number: NCT01635738), randomized to receive LP, LF, LP + LF, or a placebo for 3 months. Their Global Initiative for Asthma–based asthma severity, Childhood Asthma Control Test (C-ACT) scores, Pediatric Asthma Severity Scores, Pediatric Asthma Quality of Life Questionnaire scores, peak expiratory flow rates (PEFRs), medication use, the levels of immune biomarkers (immunoglobulin E (IgE), interferon γ, interleukin 4, and tumor necrosis factor α) at different visits, and the associated changes were evaluated. Compared with the placebo group by generalized estimating equation model, children receiving LP, LF, and LP + LF had lower asthma severity (*p* = 0.024, 0.038, and 0.007, respectively) but higher C-ACT scores (*p* = 0.005, < 0.001, and < 0.001, respectively). The LP + LF group demonstrated increased PEFR (*p* < 0.01) and decreased IgE levels (*p* < 0.05). LP, LF, or their combination (LP + LF) can aid clinical improvement in children with asthma.

## 1. Introduction

Asthma, a chronic complex disease of the airways, is characterized by reversible airflow obstruction, bronchial hyperresponsiveness, and underlying inflammation [1]. The prevalence of asthma has increased in the past decades. A potential mechanism underlying this high prevalence is the microbial hypothesis [2], which argues that less microbial exposure upregulates the cytokine production of T-helper cell type 2 (Th2), leading to an increase in allergic diseases. According to this hypothesis, probiotic administration is an alternative treatment for atopic disease, which when administered in adequate amounts, can confer a health benefit to the host [3]. The researchers found that probiotics have some health effects in atopic disease patients through immunity balancing of T-helper cell type 1 (Th1) and Th2, particularly in those with atopic dermatitis (AD). However, relevant studies focusing on asthma patients are limited.

A meta-analysis found that, although perinatal and early-life probiotic administration reduces atopic sensitization risk and total immunoglobulin E (IgE) levels in children, it may not reduce their asthma risk [4]. However, some studies have reported the benefit of using probiotics, in addition to standard care, for treating children with asthma. A randomized, placebo-controlled trial for 7-week *Enterococcus faecalis* treatment demonstrated decreased peak flow variability in children with asthma [5]. Lee et al. also reported significant improvements in the pulmonary function of children with asthma after a regimen of vegetable, fish oil, and fruit supplementation along with probiotic administration [6]. However, these aforementioned studies were designed as mixed interventions with relatively small sample sizes.

In the present study, we thus included participants representing a population of school-age children with asthma randomized to receive pure strains of *Lactobacillus paracasei* GMNL-133 (BCRC 910520, CCTCC M2011331) (LP), *Lactobacillus fermentum* GM-090 (BCRC 910259, CCTCC M204055) (LF), or their mixture (LP + LF). We focused on the therapeutic effects of the probiotics on the disease severity, quality of life, immune biomarkers, and fecal microbial composition in school-age children with asthma.

## 2. Materials and Methods

### 2.1. Participants

This double-blind, randomized, placebo-controlled trial was conducted between December 2011 and September 2013 at the pediatric outpatient clinics of Taipei Hospital, Ministry of Health and Welfare. The inclusion criteria were 6–18 years of age with a history of intermittent to moderate persistent asthma (Global Initiative for Asthma (GINA) steps 1–3) for at least 1 year. We excluded children who had received immunosuppressants, antibiotics, systemic corticosteroids, or antimycotics within 4 weeks before study enrolment or antihistamines within 3 days before study enrolment. The children who had an immunodeficiency disease, other major medical problems, or used probiotic preparations within 4 weeks before study enrolment were also excluded. We acquired written informed consent from all the parents in compliance with the principles of the Helsinki Declaration. The Taipei hospital’s Institutional Review Board ratified the study protocol (TH-IRB-10-14). The study was registered under trial number NCT01635738.

### 2.2. Protocol

**Figure 1:**
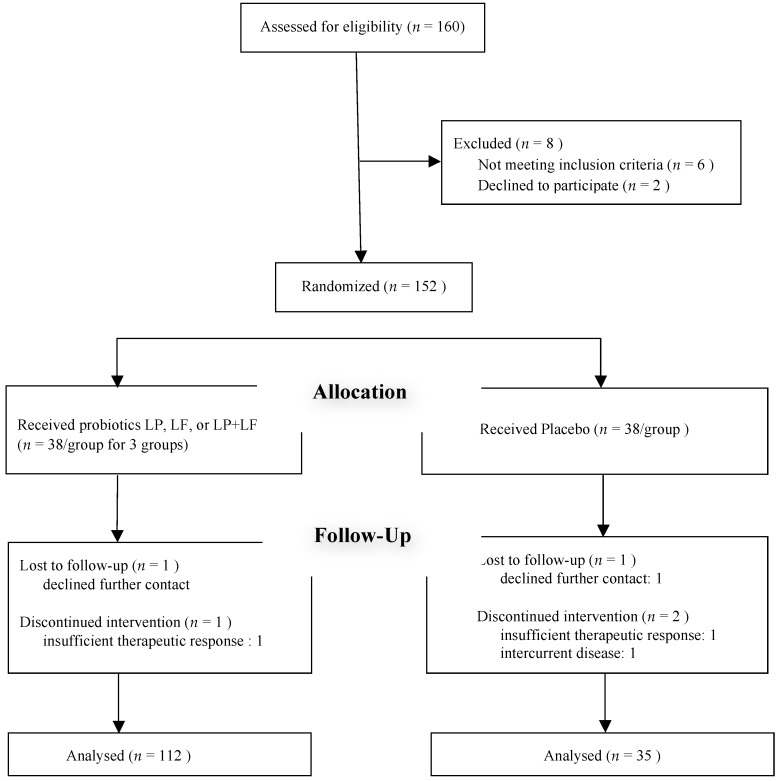
Consort diagram.

An investigator enrolled the children and sequentially assigned them a patient number associated with a code. Capsules were prepared and coded by GenMont Biotech Inc. (in their Current Good Manufacturing Practice–certified facilities, Tainan, Taiwan) and dispensed by a study nurse. The children were randomized using computer-generated 4-block design lists created by a statistician, with stratification according to age, sex, severity, and current medication use. We assessed the eligibility of 160 recruited children and randomly allocated them to four groups, with 40 participants in each group (Figure 1). The groups were then randomized to receive LP, LF, LP + LF, or placebo for 3 months. All the investigators, study nurses, and participants were blinded to treatment assignment over the study duration. A capsule count was performed monthly to ensure that the capsules were taken as applicable. Randomization code was deciphered only at the end of the trial.

### 2.3. Outcome Measures

**Figure 2:**
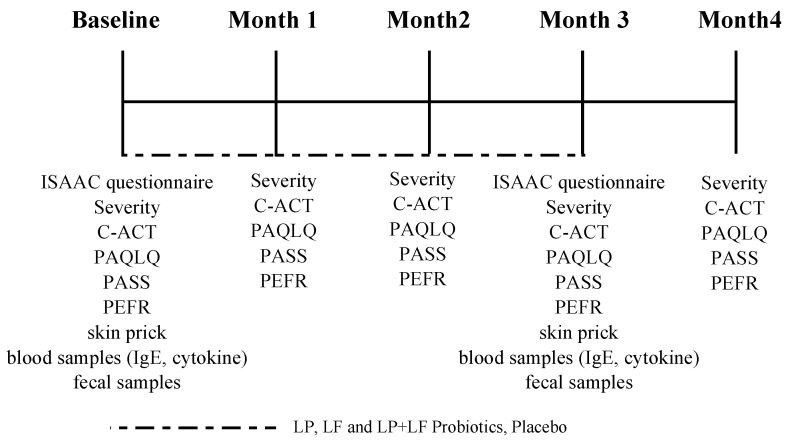
Study protocol.

The primary outcome was the changes in asthma severity and Childhood Asthma Control Test (C-ACT) scores over 3 months of the intervention compared with baseline. At baseline and follow-up visits at 0, 1, 2, 3, and 4 months of intervention, we determined GINA-based asthma severity and recorded C-ACT scores, Pediatric Asthma Quality of Life Questionnaire (PAQLQ) scores, Pediatric Asthma Severity Scores (PASSs), peak expiratory flow rates (PEFRs), and medication use. Skin prick test and blood serum analysis were performed at 0 and 3 months of intervention. In addition, fecal microbial analysis was performed for comprehensive evaluation before and after the 3-month treatment course. The changes in PAQLQ score, PASS, PEFR, skin prick test reactivity, serum immune biomarker levels, and fecal probiotic microbial composition were the secondary outcomes (Figure 2).

### 2.4. Laboratory Methods

Skin prick tests using commercial extracts were performed to detect asthma-causing allergens, including mite, cockroach, animal dander, egg, milk, and crab allergens [7]. The levels of IgE and other serum immune biomarkers, such as interferon (IFN) γ, interleukin (IL) 4, and tumor necrosis factor (TNF) α, were measured through enzyme-linked immunosorbent assay [8]. Specific intestinal bacterial strains in the feces were quantified using conventional culture techniques [9].

### 2.5. Sample Size Estimation

Using nQuery Advisor + nTerim 3.0 (Statistical Solutions Ltd., Cork, Ireland), we calculated the number of participants required to detect the presence of any significant differences in C-ACT scores. According to previous data [10], to detect significant differences in the effects of probiotics on C-ACT scores with 90% power and a 5% significance level, each study group must include at least 22 participants. To allow for a 20% loss from ineligibility or withdrawal, we decided to enroll 30 children in each group. After power assessment, we estimated that 120 total participants would suffice and thus scheduled recruitment of 160 children.

### 2.6. Statistical Analysis

The baseline demographic data of the four groups were compared using analysis of variance. Intragroup comparisons for severity, C-ACT scores, PASSs, PAQLQ scores, and immune biomarker levels at baseline and 3 months of treatment were performed using the paired *t* test. Differences in outcome variables between the treatment and placebo groups over the five visits were evaluated using a generalized estimating equation (GEE) model after adjustment for potential confounders. All children who completed the study were included in the analysis, regardless of their compliance. All reasons for dropouts or premature withdrawal from the study as well as missing values were recorded. Significance was set at a two-tailed α at 0.05, and all analyses were performed on SPSS (version 21).

## 3. Result

### 3.1. Baseline Characteristics of Participants

Of 160 recruited children, 152 were finally enrolled and randomly assigned to receive LP, LF, LP + LF, or placebo. Figure 1 shows the relevant patient consort diagram, and Table 1 presents the baseline demographic characteristics of 147 children who completed the entire evaluation. The randomization process ensured adequate comparability between the treatment and placebo groups. No statistically significant differences were observed for any demographic, clinical, or functional variables. Moreover, the clinical manifestation of asthma was similar between the two groups. No significant differences were observed in the severity, total serum IgE level, the rate of sensitization to various allergens, the PEFR, or other parameters between the groups at baseline (Table 2).

### 3.2. Effects of Probiotics on Severity of Asthma and Quality of Life 

**Figure 3:**
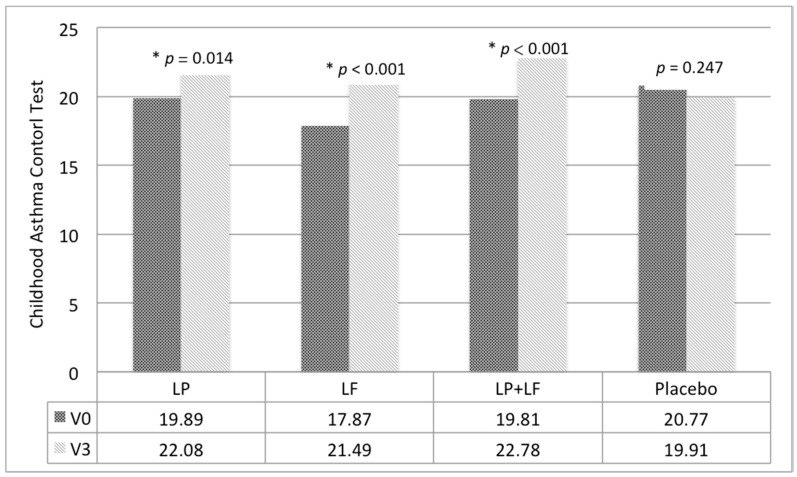
Intragroup differences in Childhood Asthma Control Test scores. * *p* < 0.05.

Significant intragroup differences were detected in C-ACT scores, the primary outcome, in all groups, except the placebo group (Figure 3). Compared with the placebo group, both asthma severity and C-ACT scores significantly improved in the LP, LF, and LP + LF groups according to our age- and sex-adjusted GEE model (Table 3). No significant difference was noted on both asthma severity and C-ACT scores between the LP + LF and the LP and LF groups, respectively. The PAQLQ scores and PASSs demonstrated no significant group-by-time effects. In the LP + LF group, PEFRs improved significantly (*p* < 0.01).

### 3.3. Effects of Probiotics on Sensitization and Immune Biomarker Levels

At the end of treatment, the total serum IgE levels significantly decreased only in the LP + LF group (*p* < 0.05; Table 4). Nevertheless, among all groups, significant intragroup differences, but no intergroup differences, were noted in the skin prick test reactivity to mite allergens. However, no such significant differences were noted for other allergens between before and after the intervention (Table S1). Moreover, serum IFN-γ, IL-4, and TNF-α levels did not demonstrate any significant changes (Table 4).

### 3.4. Fecal Microbial Composition, Rescue Medication Use and Compliance

All intervention groups showed lower counts of *Clostridium* than did the placebo group. However, these intergroup differences were nonsignificant (Table S2). The frequencies of oral steroid (Table S3), bronchodilator (Table S4), and antihistamine use (Table S5) demonstrated no significant intergroup differences. The compliance of each group is more than 93% without intergroup differences (Table S6).

## 4. Discussion

The present study effectively resolves the debate regarding whether pure probiotics are beneficial to children with asthma. Using a GEE model, we found that LP, LF, and LP + LF interventions along with standard asthma therapy effectively reduced asthma severity and increased C-ACT scores. Furthermore, LP + LF increased both serum IgE levels and PEFRs, implying the existence of a dose-dependent effect.

Consistent with the present study results, a randomized clinical trial by Chen et al. [10] revealed the beneficial effects of probiotics on the severity of asthma and clinical symptoms, but they included participants with asthma accompanied by persistent allergic rhinitis. By contrast, studies have demonstrated the significant effects of various probiotics on some parameters of asthma, but not on its clinical severity, in children with asthma [11,12]. In addition to probiotics containing a single bacterial strain, the clinical advantage of applying a mixture of bacterial strains [13] or using mixed therapy that includes a probiotic regimen [6] has been demonstrated in asthma patients. The reasons for inconsistencies in the aforementioned results may be the variabilities in genetic backgrounds, disease severity, culture, and diet of the participants; moreover, the differences in the study designs, mixed regimens, and strain doses and combinations may also affect the results because the effects of probiotics are strain-specific [14].

We selected LP for this trial because it previously demonstrated beneficial effects in airway hypersensitivity patients [15]. Moreover, it can inhibit related Th2 cytokine production and rebalance the Th1/Th2 immune response by increasing IFN-γ levels, as noted in murine bronchoalveolar lavage samples [16]. We also used LF, which can increase IFN-γ levels in patients with AD [17] and ameliorate oxidative damage, food allergies, and food-derived infections [18,19]. The present study is the first one in a clinical setting that compared the effects of both single- and mixed-strain probiotics.

Regarding the effects on serum immune biomarker levels, a clinical trial applied single-strain *Lactobacillus gasseri* A5 probiotics to school children with asthma and allergic rhinitis and reported a nonsignificant reduction in total IgE levels [10]. In the current study, the administration of LF + LP was associated with a significant decrease in IgE levels, while the administration of LP or LF alone showed a tendency to decrease IgE levels, albeit without reaching statistical significance. Similarly, the significant decrease was noted in PEFRs in the LP + LF group. These results may be explained by the synergistic interactions or dose-dependent effects, which were also noted in other probiotic-related trials [20,21]; however, the mechanisms underlying these effects remain unclear.

Studies reporting intragroup differences in the effects of probiotics on IgE levels in children with asthma are limited [22]. However, the significance of probiotic-elicited reduction in IgE levels in children with AD has been reported by several studies [23]. Notably, studies have also revealed that, in addition to regulating IgE levels, probiotics can regulate cytokine levels in patients with AD. For instance, in some AD studies [24,25], probiotics significantly reduced serum IL-4 levels, which were compatible with less Th2 responses. However, such cytokine-related findings in the context of asthma are required. The few studies mentioning the related measurement lack consistency in results [10,22].

Notably, based on the preceding comparison of different allergic diseases, gastrointestinal probiotic administration is more efficient at alleviating AD than at alleviating respiratory tract allergic diseases, namely, asthma and allergic rhinitis. Furthermore, the sensitization pattern for asthma allergens, as observed in the current study using the skin prick test, was also different from that for AD allergens. Consistent with previous studies, mites are the predominant aeroallergen of asthma [26,27,28]. We noted significant postintervention changes in mite sensitization among all groups; however, no intergroup differences were observed. Due to the difference between AD and respiratory tract allergic diseases, it might be useful to consider an alternative method for probiotic administration in children with asthma, such as the intranasal route, which demonstrated effective results in a murine model [29]; however, related human studies are warranted.

Considering the pathogenesis, asthma might be viewed as atopic processes later in life, implying a more systemic level. A review article summarized current experimental data and gave the interpretation of the allergy march; epidermal barrier dysfunction in early life was found to initiate systemic sensitization, which later facilitates the further development of asthma and allergic rhinitis [30]. Another longitudinal study suggested that children with early atopic AD have higher risk of asthma than do those with nonatopic AD [31]. Therefore, the administration of a high probiotic dose, probiotic strain mixture, or mixed therapy including probiotics appears suitable for children with asthma compared to other atopic disease; this inference is partly supported by previous trials [6,13].

Regarding fecal intestinal microbiota, the numbers of *Lactobacillus*, *Bifidobacterium*, and *Clostridium* before and after intervention demonstrated no intergroup differences among all four groups. The LP + LF group demonstrated a tendency to elevate the fecal colony counts of *Lactobacillus* and *Bifidobacterium* compared to the placebo group, but without reaching statistical significance. It may be explained by the dose-dependent effect: only the combination group received adequate probiotics against consumption. In addition to *Lactobacillus*, we analyzed the numbers of *Bifidobacterium* and *Clostridium* because asthma is associated with a higher amount of *Clostridium* [32] and a lower level of *Bifidobacterium* [33] in previous studies, respectively. We found the numbers of *Clostridium* in LP group tended to decrease after intervention, but without reaching statistical significance. This was probably because *Lactobacillus* reestablished gut microbiota, as suggested by Durack et al. [34]. The authors asserted that *Lactobacillus* supplements may recompose gut microbiota, thus preventing atopy and asthma. Furthermore, decreases in *Clostridium* counts may facilitate colonization by other microorganisms, thus increasing the gut microbial diversity, potentially another protective factor against asthma [35].

This study has some limitations. First, the children with asthma continued receiving standard therapy along with the probiotics, impeding the potential delineation of the effects of probiotics alone. However, the interference is ethical and unavoidable. Moreover, no group differences were noted in oral steroid or bronchodilator use between before and after the intervention. Additionally, these treatments may mask the therapeutic effects of probiotics, which make the results toward the null and strengthen our positive finding. Second, the compliance of the children was another concern. To ensure compliance, we used the capsule counting method rather than the patient record method; this method was easier and less expensive than detection of the study probiotic strains in the feces. Finally, some confounders, including selection bias, host factors (e.g., genetic backgrounds and original microbiota), and environmental factors (e.g., diet and lifestyle) existed. To mitigate this limitation, randomization was performed.

Nevertheless, our study has several strengths: relatively large sample size, comprehensive outcome measures (including C-ACT scores, PAQLQ scores, PASSs, and skin prick testing), and longitudinal repeated measures. We collected different types of data, specifically various serum biomarker levels and fecal microbial compositions. Moreover, a novelty of our study was that we combined two probiotic strains (LP + LF) and noted superior effects in lowering IgE and elevating PEFR than single stain alone. This topic can provide pediatricians, immunologists, and other health care professionals evidence of *Lactobacillus* administration for childhood asthma. Furthermore, it can also inspire public health experts.

## 5. Conclusions

Our study supports that *Lactobacillus* is beneficial to children with asthma. We found that both LP and LF can reduce asthma severity and improve asthma control in school-age children. The combination of LP plus LF appears to be more effective in childhood asthma than either LP or LF alone. LP, LF, and their combination were well tolerated with fair compliance and without adverse effects reported.

## Figures and Tables

**Table 1:** Baseline demographic characteristics of study participants (*N* = 147).

Characteristics	LP (*n* = 38)	LF (*n* = 38)	LP + LF (*n* = 36)	Placebo Group (*n* = 35)
Male, *N* (%)	22 (57.9)	24 (63.2)	19 (52.8)	18 (51.4)
Age (years), Mean (SD)	7.68 (2.21)	7.37 (2.34)	7.00 (1.79)	7.86 (2.50)
Height (cm), Mean (SD)	121.24 (18.49)	117.81 (17.21)	117.51 (15.89)	122.23 (18.94)
Weight (Kg), Mean (SD)	26.45 (10.08)	25.14 (10.16)	24.75 (10.29)	26.30 (11.76)
IgE (kU/I), Mean (SD)	611.26 (511.83)	600.23 (739.18)	748.22 (896.40)	493.06 (773.52)
Combine with allergic rhinitis (AR), *N* (%)	31 (81.6)	31 (81.6)	32 (88.9)	28 (80.0)
Combine with atopic dermatitis (AD), *N* (%)	15 (39.5)	10 (26.3)	11 (30.6)	9 (25.7)
Allergic sensitization, *N* (%)				
Mite	33 (86.8)	32 (84.2)	30 (83.3)	29 (82.9)
Cockroach	3 (7.9)	2 (5.3)	1 (2.8)	0 (0)
Animal dander	2 (5.3)	2 (5.3)	1 (2.8)	1 (2.9)
Milk	2 (5.3)	3 (7.9)	1 (2.8)	0 (0)
Egg	2 (5.3)	2 (5.3)	1 (2.8)	1 (2.9)
Crab	2 (5.3)	3 (7.9)	1 (2.8)	0 (0)
Maternal history of atopic disease, *N* (%)	21 (55.3)	22 (57.9)	12 (33.3)	15 (42.9)
Paternal history of atopic disease, *N* (%)	21 (55.3)	21 (55.3)	20 (55.6)	17 (48.6)

LP, *Lactobacillus paracasei*; LF, *Lactobacillus fermentum*.

**Table 2:** Subscale measures at baseline (*N* = 147).

Subscale	LP (*n =* 38) Mean (SD)	LF (*n* = 38) Mean (SD)	LP + LF (*n* = 36) Mean (SD)	Placebo (*n* = 35) Mean (SD)	*p*-Value 4 Groups
Severity	2.16 (0.64)	2.13 (0.62)	2.28 (0.62)	2.20 (0.58)	0.757
C-ACT	19.89 (4.28)	17.87 (5.54)	19.81 (4.72)	20.77 (4.75)	0.074
PAQLQ	5.44 (1.17)	5.40 (1.41)	5.90 (0.85)	5.58 (1.16)	0.268
PASS	16.84 (5.27)	16.29 (5.79)	15.71 (4.85)	15.60 (5.16)	0.738
IFN-γ (ng/uL)	170.73 (188.44)	150.75 (158.12)	156.97 (167.18)	145.28 (162.26)	0.954
IL-4 (ng/uL)	36.05 (29.63)	48.48 (85.06)	61.10 (117.67)	55.38 (86.94)	0.773
TNF-α (ng/uL)	292.95 (425.14)	324.71 (512.82)	231.88 (187.97)	207.51 (340.37)	0.690
IgE (kU/I)	611.26 (511.83)	600.23 (739.18)	748.22 (896.40)	493.06 (773.52)	0.547
Fecal cell count Log10 (CFU/g)					
*Lactobacillus*	8.07 (0.87)	7.90 (0.95)	7.49 (1.15)	7.71 (0.92)	0.101
*Bifidobacterium*	8.81 (0.91)	8.74 (0.85)	8.75 (1.12)	8.90 (0.72)	0.872
*Clostridium*	7.19 (0.78)	6.63 (1.09)	6.84 (1.18)	6.79 (1.11)	0.133

LP, *Lactobacillus paracasei*; LF, *Lactobacillus fermentum*; C-ACT, Childhood Asthma Control Test; PAQLQ, Pediatric Asthma Quality of Life Questionnaire; PASS, Pediatric Asthma Severity Scores.

**Table 3:** The *p* values and effect sizes (β) compared with the placebo group (*n* = 35) using a generalized estimating equation model.

Measure	LP Group (*n* = 38)		LF Group (*n* = 38)		LP + LF Group (*n* = 36)	
	β (95% CI)	*P* Value	β (95% CI)	*P* Value	β (95% CI)	*P* Value
Severity	−0.34 (−0.63, −0.04)	0.024 *	−0.30 (−-0.59, −0.02)	0.038 *	−0.43 (−0.74, −0.12)	0.007 *
C-ACT	3.13 (−0.95, 5.31)	0.005 *	4.54 (2.44, 6.65)	< 0.001 *	3.83 (1.78, 5.89)	< 0.001 *
PAQLQ	−0.32 (−0.86, 0.23)	0.256	−0.30 (−0.89, 0.28)	0.310	−0.43 (−0.96, 0.09)	0.104
PASS	−1.39 (−4.09, 1.30)	0.311	−1.14 (−3.79, 1.51)	0.400	−0.48 (−3.17, 2.20)	0.725
PEFR	8.77 (−11.74, 29.27)	0.402	28.83 (−3.55, 61.21)	0.081	33.81 (8.62, 59.00)	0.009 *

Adjusted for age and sex, * *p* < 0.05. LP, *Lactobacillus paracasei*; LF, *Lactobacillus fermentum*; C-ACT, Childhood Asthma Control Test; PAQLQ, Pediatric Asthma Quality of Life Questionnaire; PASS, Pediatric Asthma Severity Scores; PEFR, peak expiratory flow rate.

**Table 4:** Immune biomarker levels at baseline and subsequent changes (*N* = 147).

Subscale	Examination	LP (*n* = 38) Mean (SD)	LF (*n* = 38) Mean (SD)	LP + LF (*n* = 36) Mean (SD)	Placebo Group(*n* = 35) Mean (SD)	*p*-Value 4 Groups
IgE	Baseline	611.26 (511.83)	600.23 (739.18)	748.22 (896.40)	493.06 (773.52)	0.547
(kU/I)	month 3	482.42 (371.68)	496.40 (622.51)	377.29 (268.51) *	577.81 (705.94)	0.448
IFN-γ	Baseline	170.73 (188.44)	150.75 (158.12)	156.97 (167.18)	145.28 (162.26)	0.954
(ng/uL)	month 3	158.01 (164.61)	182.75 (373.50)	221.41 (426.21)	166.21 (197.61)	0.886
IL-4	Baseline	36.05 (29.63)	48.4 (85.06)	61.10 (117.67)	55.38 (86.94)	0.773
(ng/uL)	month 3	51.16 (85.33)	49.58 (67.82)	55.09 (109.40)	104.13 (215.39)	0.366
TNF-α	Baseline	292.95 (425.14)	324.71 (512.82)	231.88 (187.97)	207.51 (340.37)	0.690
(ng/uL)	month 3	315.23 (625.85)	777.92 (1731.71)	584.30 (959.98)	550.48 (1059.83)	0.514

LP, *Lactobacillus paracasei*; LF, *Lactobacillus fermentum*. * *p* < 0.05 (Intragroup comparisons).

## Supplementary Materials

The following are available online at http://www.mdpi.com/2072-6643/10/11/1678/s1, Table S1: Skin prick test reactivity, Table S2: Fecal bacterial colony counts, Table S3: Oral steroid use frequency at baseline and during follow-up visits, Table S4: Oral bronchodilator use frequency at baseline and during follow-up visits, Table S5: Oral anti-histamine use frequency at baseline examination and on follow-up visits, Table S6: Probiotics capsules mean taken days monthly record.
