# Supplementary material for: Efficacy of Lactobacillus Administration in School-Age Children with Asthma: A Randomized, Placebo-Controlled Trial
Source: Nutrients. 2018 Nov 5;10(11):1678. doi: 10.3390/nu10111678 (PMC6265750; doi:10.3390/nu10111678)
Supplement: Supplementary file 1 [file nutrients-10-01678-s001.pdf]

Table S1. Skin prick test reactivity.

	Examination	LP (n = 38) Mean (SD)	LF (n = 38) Mean (SD)	LP + LF (n = 36) Mean (SD)	Placebo Group (n = 35) Mean (SD)	p-Value 4 Groups
Mite	Baseline	2.55 (0.89)	2.55 (0.92)	2.31 (0.98)	2.34 (1.06)	0.553
	month 3	1.61 (0.61) *	1.61 (0.84) *	1.45 (0.77) *	1.43 (0.70) *	0.614
Cockroach	Baseline	0.62 (0.87)	0.73 (1.28)	0.45 (0.69)	0.33 (0.52)	0.821
	month 3	0.10 (0.32)	0.00 (0.00)	0.09 (0.30)	0.0 (0.00)	0.683
Animal dander	Baseline	0.46 (0.78)	0.45 (0.82)	0.45 (0.69)	0.60 (0.89)	0.985
	month 3	0.10 (0.32)	0.00 (0.00)	0.09 (0.30)	0.17 (0.41)	0.701
Milk	Baseline	0.54 (0.78)	0.73 (0.91)	0.36 (0.67)	0.33 (0.52)	0.650
	month 3	0.10 (0.32)	0.00 (0.00)	0.09 (0.30)	0.00 (0.00)	0.683
Egg	Baseline	0.54 (0.78)	0.55 (0.82)	0.36 (0.67)	0.60 (0.89)	0.920
	month 3	0.10 (0.32)	0.00 (0.00)	0.09 (0.30)	0.00 (0.00)	0.683
Crab	Baseline	0.46 (0.78)	0.55 (0.93)	0.55 (0.69)	0.50 (0.55)	0.992
	month 3	0.10 (0.32)	0.00 (0.00)	0.09 (0.30)	0.00 (0.00)	0.683

LP, *Lactobacillus paracasei*; LF, *Lactobacillus fermentum*. *$p < 0.05$ (Intragroup comparisons).

Table S2. Fecal bacterial colony counts.

	Examination	LP (n = 38) Mean (SD)	LF (n = 38) Mean (SD)	LP + LF (n = 36) Mean (SD)	Placebo Group (n = 35) Mean (SD)	p-Value 4 Groups
Lactobacillus	Baseline	8.07 (0.87)	7.90 (0.95)	7.49 (1.15)	7.71 (0.92)	0.101
(log [CFU/g])	month 3	7.55 (0.77)	7.84 (0.98)	7.95 (1.01)	7.51 (1.31)	0.267
Bifidobacterium	Baseline	8.81 (0.91)	8.74 (0.85)	8.75 (1.12)	8.90 (0.72)	0.872
(log [CFU/g])	month 3	8.49 (1.10)	8.74 (1.08)	8.93 (0.81)	8.70 (1.35)	0.518
Clostridium	Baseline	7.19 (0.78)	6.63 (1.09)	6.84 (1.18)	6.79 (1.11)	0.133
(log [CFU/g])	month 3	6.65 (1.27)	6.56 (0.92)	6.84 (1.14)	6.88 (1.30)	0.632

LP, *Lactobacillus paracasei*; LF, *Lactobacillus fermentum*.

Table S3. Oral steroid use frequency at baseline and during follow-up visits.

Frequency (Mean times/month)	LP (n = 38)	LF (n = 38)	LP + LF (n = 36)	Placebo Group (n = 35)	p-Value 4 Groups
Baseline	2.05	3.03	1.81	2.31	0.811
Month 1	0.32	0.29	0.08	0.57	0.297
Month 2	0.32	0.22	0.62	0.53	0.752
Month 3	0.09	0.53	0.18	0.63	0.242
Month 4	0.26	0.42	0.21	0.33	0.874

Table S4. Oral bronchodilator use frequency at baseline and during follow-up visits.

Frequency (Mean times/month)	LP (n = 38)	LF (n = 38)	LP + LF (n = 36)	Placebo Group (n = 35)	p-Value 4 Groups
Baseline	2.97	3.13	2.17	4.43	0.677
Month 1	0.95	1.82	1.25	1.34	0.574
Month 2	1.11	1.05	1.74	1.47	0.777
Month 3	0.49	0.67	1.18	1.63	0.273
Month 4	0.64	0.56	0.50	0.18	0.753

Table S5. Oral anti-histamine use frequency at baseline examination and on follow-up visits.

Frequency (Mean times/month)	LP (n = 38)	LF (n = 38)	LP + LF (n = 36)	Placebo Group (n = 35)	p-Value 4 Groups
Baseline	3.08	3.00	4.67	1.67	0.878
Month 1	1.08	0.00	0.67	0.33	0.464
Month 2	1.69	1.70	0.55	1.40	0.705
Month 3	0.00	0.50	0.18	0.00	0.303
Month 4	0.00	0.00	0.00	1.80	0.079

Table S6. Probiotics capsules mean taken days monthly record.

Mean Taken Days	LP (n = 38)	LF (n = 38)	LP + LF (n = 36)	Placebo Group (n = 35)	p-Value 4 Groups
Month 1	28.6	28.7	28.1	28.6	0.286
Month 2	28.7	28.7	28.7	28.6	0.992
Month 3	29.1	28.4	28.4	28.8	0.756
